# Composition of Taurine

**Published:** 1846-09

**Authors:** J. Redtenbacher


*Composition of Taurine.* By Prof. J. Redtenbacher.—Demarçay was the first who made an elementary analysis of taurine, which was discovered by L. Gmelin in 1824 ; he advanced for it the formula $C^4NH^7O^{10}$, which was confirmed by Pelouze and Dumas. Gmelin had observed that taurine dissolved in sulphuric acid without alteration, as well as that nitric acid had no effect on it, which observation the author has also repeated. However, it was highly improbable that a neutral body containing 63 per cent. oxygen should in no way yield products of decomposition which might throw light on its nature. The author therefore conveyed some taurine into potash in a state of fusion, and decomposed the melted and cold mass with dilute sulphuric acid, when a considerable quantity of sulphurous acid was disengaged. The materials employed were pure ; the sulphurous acid could therefore only arise from the taurine. On ignition upon platinum foil, the carbonized taurine likewise gave off sulphurous acid, thus placing beyond all doubt the presence of sulphur in taurine.

Nevertheless the quantitative estimation of the sulphur presents great difficulty; for it is so intimately combined in the taurine, that it can be determined neither by nitric acid nor by *aqua regia*, but only by combustion with nitre. Since now, on igniting the taurine in a platinum crucible with nitrate of soda and carbonate of barytes, an excess of sulphur was obtained, and on burning it with nitrate and carbonate of soda a loss resulted, it was ignited in a glass tube. The weighed quantity of taurine was mixed with a powdered quantity of nitrate and carbonate of soda in the mortar, transferred into the tube as in organic analysis, and carefully burnt. The residuary saline mass, together with the pieces of the tube which had cracked on cooling, was boiled with muriatic acid and water, the liquid evaporated to dryness, the silicic acid separated by filtration, and the sulphuric acid precipitated from the filtered solution with a salt of barytes. In this way four experiments gave on an average 25·70 per cent. sulphur.

As however this hitherto unknown amount of sulphur might be the cause of the relations of the other elements of taurine being erroneous, the author submitted it to analysis:—

| | Redtenbacher. | Demarçay. | Pel. and Dumas. | | |
|---|---|---|---|---|---|
| Carbon . . . . . | 19·28 | 19·27 | 18·90 | = 300·0 | 19·2 |
| Nitrogen . . . . | 11·25 | 11·20 | 11·19 | 175·0 | 11·2 |
| Hydrogen* . . . | 5·73 | 5·67 | 5·66 | 87·5 | 5·6 |
| Oxygen . . . . . | 38·04 | . . | . . | 600·0 | 38·4 |
| Sulphur . . . . . | 25·70 | . . | . . | 400·0 | 25·6 |

Taurine, $C^4NH^7O^6S^2$, is accordingly a substance most rich in sulphur; and as it is a product of decomposition of the bile, this itself must be a sulphurous body, and the ideas respecting the function of the bile be consequently modified. In a body so neutral as taurine, which resists nearly all reagents, it is difficult to decide in what way the sulphur is combined. On treating a solution of taurine with nitrate of silver and ammonia, not the least reaction occurs any more than on boiling with barytic water. If however the sulphur were contained in it in the state of sulphuric acid, a precipitate would certainly result in the barytic water. As already observed, neither nitric nor nitromuriatic acid acts upon taurine, nor even dry chlorine; but if it be heated to decomposition in the bulb of a reduction-tube, whilst a current of dry chlorine gas is passed over it, a small quantity of a liquid is obtained, in which sulphuric acid may be detected. If taurine be oxidized with caustic potash, and the residuary saline mass decomposed with dilute sulphuric acid, sulphuretted hydrogen and sulphurous acid are evolved, and sulphur separates precisely as if the potash had been treated with pure sulphur.—*Chem. Gazette, June* 15, 1846, *from Liebig's Annalen*, lvii. p. 170.

*We have corrected an error in this table, as printed in the Chemical Gazette, in which the nitrogen and hydrogen are transposed.—*Ed.*

